# pH-Sensitive Hydrogel Bilayers: Investigation on Transient Swelling-Induced Bending through Analytical and FEM Approaches

**DOI:** 10.3390/gels9070563

**Published:** 2023-07-10

**Authors:** Mahdi Askari-Sedeh, Mostafa Baghani

**Affiliations:** School of Mechanical Engineering, College of Engineering, University of Tehran, Tehran 14399-57131, Iran

**Keywords:** pH-responsive hydrogel, swelling kinetics, non-linear bending, analytical modeling, bilayer structure

## Abstract

pH-responsive hydrogels are recognized as versatile sensors and actuators due to their unique time-dependent properties. Specifically, pH-sensitive hydrogel-based bilayers exhibit remarkable bending capabilities when exposed to pH-triggered swelling. This study introduces a semi-analytical technique that combines non-linear solid mechanics with ionic species transport to investigate the bending behavior of such bilayers. The technique is validated through numerical simulations, exploring the influence of kinetic and geometric properties on bilayer behavior. The results highlight the significance of the interfacial region, particularly in configurations with lower hydrogel geometric ratios, which are susceptible to rupture. The study also uncovers the benefits of a lower hydrogel layer ratio in improving the swelling rate and final deflection, with a stronger effect observed in the presence of a buffer solution. Additionally, the compressibility of the elastomer contributes to the durability of the final bent shape. These findings enhance our understanding of pH-sensitive hydrogel-based bilayers and offer valuable insights for their design and optimization in diverse applications.

## 1. Introduction

Responsive bilayers encompass many materials and hold significant promise for diverse applications. These bilayers possess the remarkable ability to adapt and respond to external stimuli, making them highly versatile in various fields. Examples of responsive bilayers include hydrogel-based bilayers [1], shape memory polymer (SMP)-based bilayers [2], and numerous other types that continue to be explored. In the case of hydrogel-based bilayers, hydrogels offer superior biocompatibility, providing enhanced encapsulation and protection for sensitive molecules. Additionally, hydrogels demonstrate easy fabrication and stimulus responsiveness, distinguishing them from other carrier systems. The unique advantages of hydrogels make them highly attractive for diverse applications, particularly in medicine and tissue engineering. Hydrogel-based bilayers, with their high water content and customizable characteristics, find applications in biomedicine [3] and muscle-like actuators [4] while also demonstrating the ability to mimic biological membranes. Recent advances in synthesizing and characterizing hydrogel-based bilayers have demonstrated their potential in various applications. As an illustration, Luneva et al. [5] conducted a study exploring the use of a bilayer hydrogel that responds to pH to facilitate wound healing.

One of the primary stages in exploring hydrogels involves formulating theories that describe their behavior. The classical theory of swelling established the mechanisms of swelling in neutral hydrogels by applying the widely recognized polymer solution theory, commonly referred to as the Flory–Huggins theory [6]. Furthermore, scientists have investigated the triggers that can prompt swelling behavior in hydrogels. The primary stimuli include temperature [6], pH [7], magnetic field [8], and light [9]. Examples include temperature-sensitive hydrogels, such as Poly(ethylene glycol) (PEG) [10], which undergo reversible swelling in response to temperature changes, enabling their use in wound healing and drug delivery applications. Conversely, pH-sensitive hydrogels such as Poly(acrylic acid) or PAA [11] respond to pH variations, swelling reversibly and making them useful for biosensing applications.

To gain a thorough understanding of hydrogel behavior and attributes, it is essential to use models of hydrogel swelling. Two swelling models are commonly used: steady-state models [9,12,13] and transient models [6,14]. While steady-state models describe a hydrogel at equilibrium, where its behavior is assumed to be independent of time, this assumption may not always hold for hydrogel swelling. Hydrogels can exhibit time-dependent deformation behavior that changes over time, and steady-state models may not adequately capture this behavior. In contrast, transient models account for swelling kinetics. The process of diffusion through hydrogel swelling is intricate and dependent on time. As a result, modeling hydrogel behavior at equilibrium may not always effectively capture the characteristics of hydrogel deformation behavior critical to their performance [6]. Studies have recently focused on incorporating hydrogel kinetics into their theories to produce more accurate predictions of time-dependent swelling behavior [6].

Considerable attention has been given to the analysis of the finite deformation of hydrogels, including the bending of a block [15,16], torsion, extension, and inflation of hydrogel cylinders [17], inflation of a cylinder [18] and spherical membrane [19,20], have been carried out. One of particular interest in studying hydrogel deformation behaviors is the study of hydrogel-based bilayers and their finite bending triggered by swelling [21]. The investigation holds promise for a variety of soft robotic applications, such as grippers [22], actuators [23,24], and self-folding structures [25,26]. Morimoto and Ashida [27] examined the steady-state bending behavior of a temperature-sensitive hydrogel-based bilayer under finite conditions by applying non-linear continuum mechanics theories. Their study has uncovered an exact solution for the finite bending of hyperelastic rectangular beams, which involves utilizing a deformable gradient breakdown through two distinct regimes of deformation, namely swelling and bending. Similarly, Abdolahi et al. [28] created a partially analytical approach to determine the finite bending of hydrogel-based bilayers triggered by swelling under varying temperatures. Moreover, They utilized a similar methodology to model the bending behavior of bilayer hydrogels composed of pH-sensitive hydrogels [29].

Understanding how pH-responsive hydrogel bilayers bend is crucial for designing bilayer structures in soft robotics and biomedical engineering. Previous models have limitations in capturing transient behavior [29] and large deformations [30]. The present research explicitly addresses the kinetics of swelling during transient bending. This model offers several notable advantages compared to previous models, including:

1. Improved drug delivery prediction: A time-dependent model can accurately capture pH-sensitive hydrogel swelling kinetics, crucial for drug release timing and rate;
2. Improved mechanical stability understanding: Large-deformation theory allows insights into pH-sensitive hydrogel durability under substantial deformations. The model can assess the hydrogel’s response to real-world mechanical forces, aiding in designing hydrogels for specific applications, such as tissue engineering or responsive coatings;
3. The bidirectional coupling of chemo-mechanical fields: Considering the influence of transient chemical kinetics on mechanical deformation, they offer great potential in advancing our understanding of mechanical phenomena such as creep and viscoelasticity, particularly in tough hydrogels.

## 2. Results

This section uses the proposed semi-analytical theory to investigate a constrained cylindrical swelling behavior responsive to pH stimuli. The theory’s predictions are validated against the experimental findings of De et al. [31]. Additionally, the theory is employed to analyze the bending deformation of a hydrogel bilayer caused by pH-stimuli swelling. To validate the theory, numerical simulations using COMSOL Multiphysics software are conducted. Although COMSOL Multiphysics is highly flexible and can simulate a wide range of problems, the numerical challenges associated with FEM simulations make it a time-consuming process compared to analytical procedures.

### 2.1. Experimental Verification of the Theory’s Predictions

**Figure 1:**
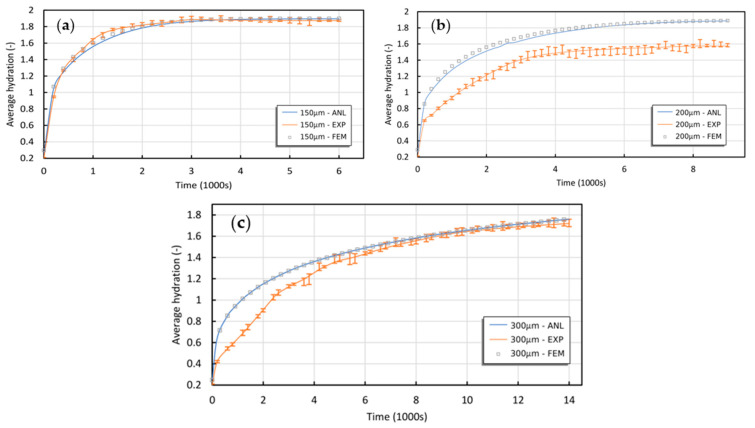
Comparing the presented semi-analytical model to experiments and 3D FEM simulations for transient swelling of the pH-sensitive solid cylinder with diameters of (**a**) 150 μm, (**b**) 200 μm, and (**c**) 300 μm exposed to pH stimuli [17].

**Figure 2:**
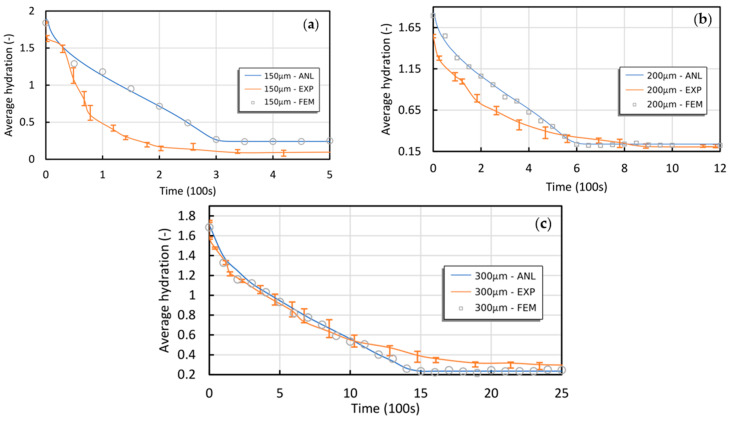
Comparing the presented semi-analytical model to experiments and 3D FEM simulations for transient de-swelling of the pH-sensitive solid cylinder with diameters of (**a**) 150 μm, (**b**) 200 μm, and (**c**) 300 μm exposed to pH stimuli [17].

Experimental data obtained by De, Aluru, Johnson, Crone, Beebe, and Moore [31] were compared to the suggested semi-analytical theory, as illustrated in Figure 1 and Figure 2. The experiment involved a solid cylindrical hydrogel responsive to pH stimuli with various diameters (D = 150, 200, and 300 μm) confined by a 1000 μm to 180 μm glass channel on both ends. The hydrogel was exposed to environmental stimuli of pH from 3 to 6.

The 3D FEM simulations and experimental tests exhibited a significant level of agreement with the semi-analytical model, with one exception: the 200 μm sample. In this case, the noticeable increase in error bars over time may be attributed to potential data acquisition issues rather than inherent variations and complexities within the system. Nonetheless, the semi-analytical model predicts that the equilibrium hydration of the 200 μm sample should fall between that of the 150 μm and 300 μm samples.

The deswelling experiments revealed that hydrogels undergo dehydration at varying rates, characterized by an initial phase of significant dehydration, slower deswelling, and eventually severe deswelling again. The proposed semi-analytical model demonstrates greater accuracy in predicting this behavior than the previous model developed by De, Aluru, Johnson, Crone, Beebe, and Moore [31]. It is important to note that during the deswelling process, the hydrogel is expected to return from the previous equilibrium state to its initial hydration ($H_{0}=0.2$). However, this behavior was violated in the 150 μm experiments, where measurement errors may have contributed to the inconsistency observed in the error bars of this particular case.

### 2.2. Modeling of the Hydrogel-Based Bilayer Bending

**Figure 3:**
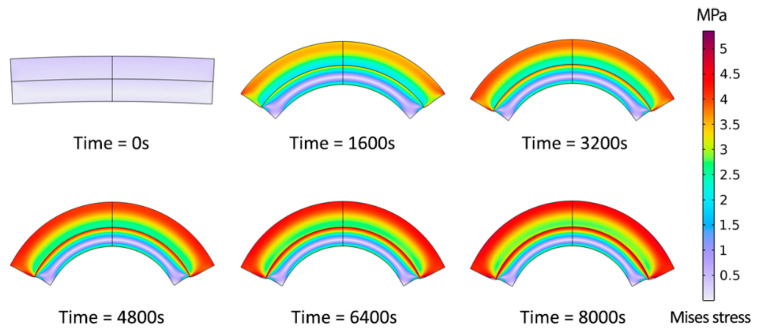
Numerical modeling of bilayer bending resulting from pH-triggered swelling.

A hydrogel-based bilayer of 300 μm length with each layer of 50 μm thickness, as shown in Figure 3, using a mesh of 1200 quadrilateral elements, was considered. While the analytical approach required no constraints, the FEM model required appropriate boundary conditions for the two ends of the beam. The same chemical potential boundary conditions were applied to the ends used for the outer radius, replicating actual conditions. To avoid rigid body motion issues, the bilayer’s midline perpendicular to the interface was considered axisymmetric, and the midpoint of the interface was fixed to ensure that the hydrogel layer’s swelling was not affected.

**Figure 4:**
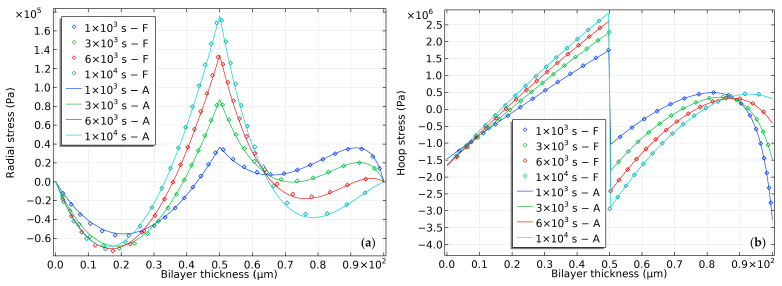
Stress distribution in a semi-analytical model of bilayer bending resulting from pH-triggered swelling. (**a**) Radial stress distribution and (**b**) hoop stress distribution.

The data presented in Figure 4 illustrate the distribution of radial and hoop stresses that occur during the swelling process. The stress profile exhibits noteworthy non-linearities due to variations in pH. In the early stages, the outermost surface experiences significant negative hoop stress, attributed to the osmotic pressure resulting from a sudden decrease in the ion profile. This osmotic pressure is treated as external stress, which is subtracted from the lateral stress. As the process continues, the net hoop stress progressively increases and is counteracted by the lateral tension stress of the network.

Furthermore, it is observed that the compressive hoop stress within the inner surface of the elastomer remains relatively stable, in contrast to the significant fluctuations experienced by the outer surface. This may indicate that the elastomer is not maintaining its volume through compressibility. Nevertheless, at the interface of the bilayer, there is a noticeable increase in the difference in hoop stress, potentially resulting in the rupture of the hydrogel and elastomer materials.

**Figure 5:**
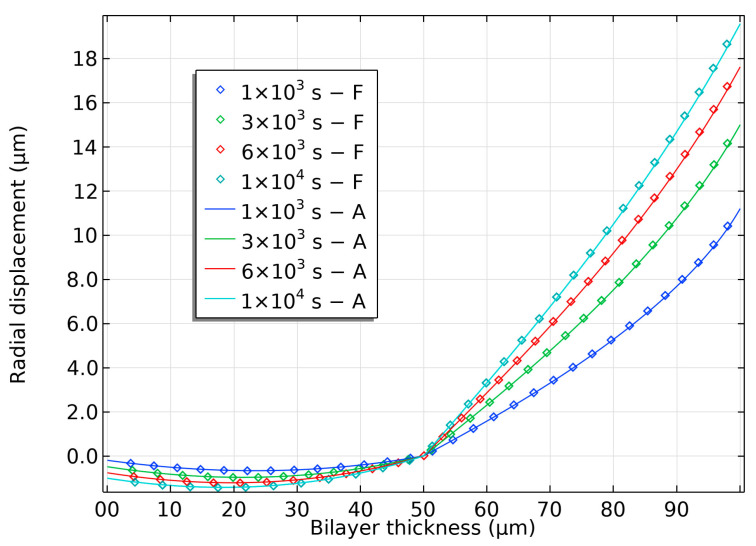
Radial displacement in semi-analytical bilayer bending resulting from pH-triggered swelling.

The radial displacement of the bilayer at various times is shown in Figure 5. During swelling, the hydrogel layer experiences an increase in radial displacement, while the elastomer layer undergoes compressive radial displacement. The tangential stretch produced by the hydrogel layer induces the contraction of the elastomer layer, which is evident in its radial displacement. Nonetheless, the elastomer’s compressibility limits this contraction, resulting in less tendency for the bilayer to recover its flat shape.

In the early stages of swelling, the outer region of the hydrogel experiences non-linear radial displacement due to its proximity to the surrounding solvent and faster diffusion. However, as the hydrogel approaches its fully swollen state, the radial displacement along the thickness becomes linear, and the non-linear effects fade.

**Figure 6:**
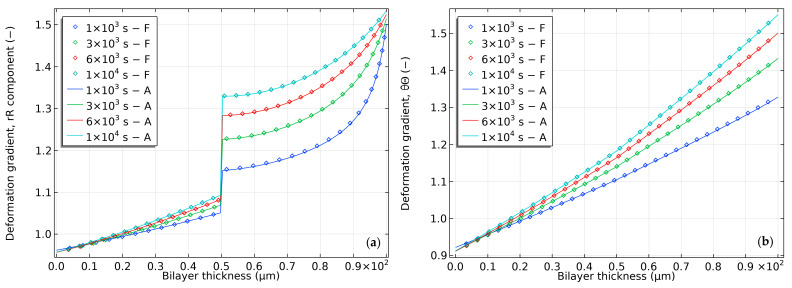
Deformation gradient distribution in a semi-analytical model of bilayer bending resulting from pH-triggered swelling. (**a**) Radial component distribution and (**b**) tangential component distribution.

Figure 6 shows the radial and tangential components of the deformation gradient in the hydrogel-based bilayer. The outer surface reaches its equilibrium deformation, eventually propagating to deeper radial deformations. However, bending deformation causes radii of curvature below the neutral axis to remain smaller than those above it. The tangential component reveals increasing non-uniformity in the bilayer interface, characterized by a gradual increase in the slope of the hydrogel layer relative to the elastomer layer.

**Figure 7:**
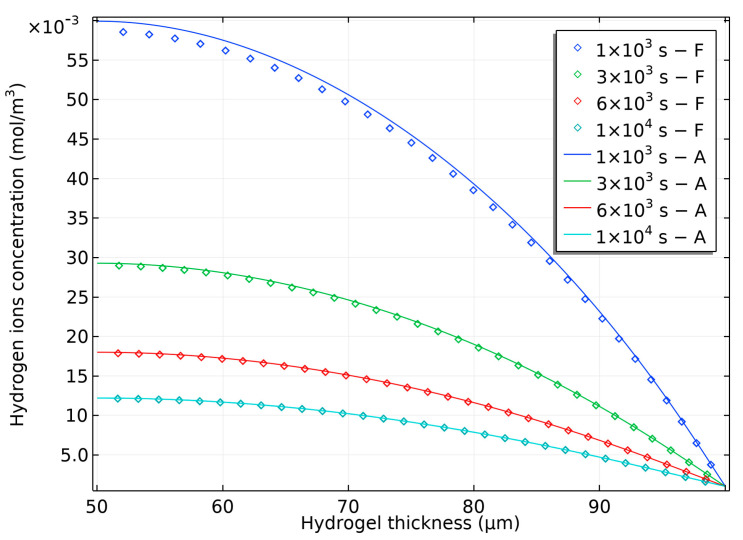
Hydrogen ion’s distribution in semi-analytical bilayer bending resulting from pH-triggered swelling.

The hydrogen ions concentration at different times is shown in Figure 7. Initially, there is a significant difference between the ion concentration in the bilayer and surroundings, resulting in a rapid change in concentration across the thickness. The ion distribution gradually becomes more uniform and reaches a plateau. It is worth noting that due to binding to the elastomer, the flux ion at the interface remains zero throughout the process.

**Figure 8:**
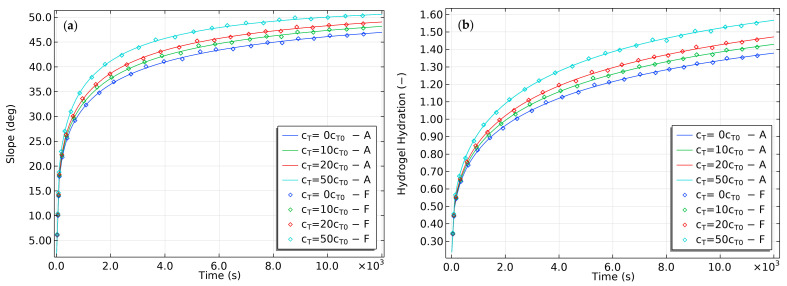
The effect of varying buffer concentrations on the (**a**) slope and (**b**) hydration of the bilayer bending resulting from pH-triggered swelling.

During free swelling experiments with gels, researchers have noted that the swelling rate is faster in buffered solutions that help maintain a constant pH [32]. This observation suggests that pH can significantly affect the swelling behavior of gels, which could have implications for applications that require precise control of the swelling rate. To investigate the effect of buffer concentration, the current study varied the buffer concentration and recorded the results in Figure 8. Increasing the buffer concentration did not significantly alter the final slope of the bilayer, unlike the final hydration. However, it did reduce the time needed to reach the final slope and the corresponding equilibrium hydration. These findings indicate that buffer concentration can be manipulated to control the gels’ swelling rate, which is potentially useful in drug delivery systems and other applications that require controlled swelling behavior.

**Figure 9:**
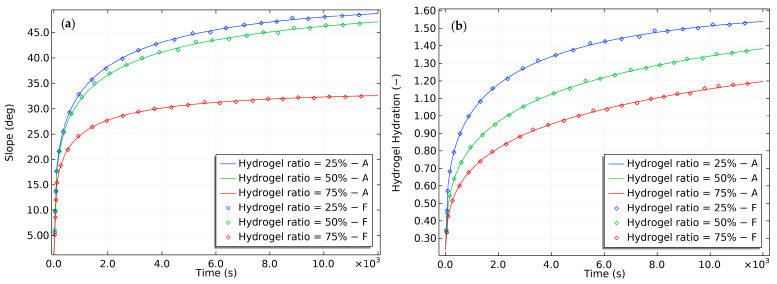
The effect of hydrogel thickness on the (**a**) slope and (**b**) hydration of the bilayer bending resulting from pH-triggered swelling.

The diffusion process in hydrogels resembles heat transfer and is significantly influenced by the dimensions of the structure. The time needed for a hydrogel component with specific material properties to swell fully is proportional to its size. Figure 9 illustrates this effect, showing that the slope of a bilayer containing 75% hydrogel thickness experiences a significant reduction in its slope and requires approximately 4000 s to reach equilibrium. However, for bilayers composed of 25% and 50% hydrogel, the slope and time required to reach equilibrium do not vary significantly.

**Figure 10:**
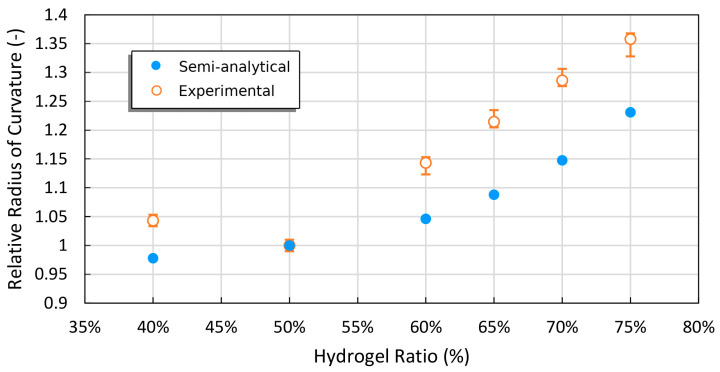
The effect of hydrogel thickness on the radius of curvature increases.

The proposed model investigates the increase of radius of curvature with the 50% hydrogel ratio as a reference, as shown in Figure 10, taking into account the layer ratio effect observed in previous experiments conducted by Kim et al. [33].

**Figure 11:**
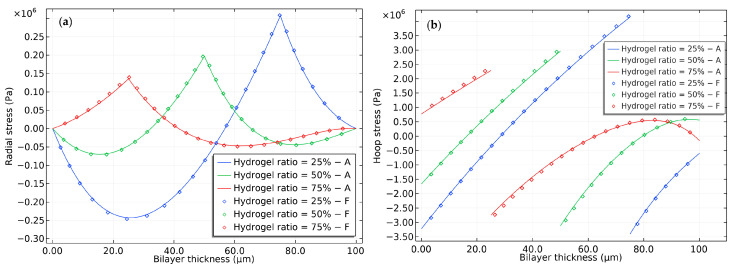
The hydrogel ratio on the (**a**) radial and (**b**) hoop stress distribution of the bilayer bending resulting from pH-triggered swelling.

Figure 11 provides valuable insights into the relationship between hydrogel thickness and stress. The results demonstrate that as the hydrogel thickness decreases, the discontinuity of tangential stress increases, which can lead to interface rupture. The compressive tangential stress on the inner surface of the hydrogel indicates that the hydrogel tends to elongate longitudinally. However, this deformation is prevented by the outer surface of the elastomer. As a result, an increase in the length of the hydrogel results in increased tension and compression at the interface. These findings have important implications for the design of hydrogel-based materials and devices, where maintaining system integrity and performance is essential. At a thickness of 75%, the radial and hoop stress at the outer surface of the bilayer hydrogel is relatively small, indicating that this region behaves more like a free hydrogel layer immersed in the solvent. At this specific thickness, the inner surface of the bilayer hydrogel exhibits tensile tangential stress, indicating that the pH stimulus induces predominantly longitudinal tension rather than bending deformation.

## 3. Conclusions

The objective was to develop a chemo-mechanical model that could predict the bending behavior of bilayers composed of hydrogel materials, which respond to changes in environmental pH by undergoing transient swelling. The proposed model was validated against experimentally constrained swelling and finite element simulations, demonstrating good agreement. The model consists of a partial and an ordinary differential equation coupled together, where the former describes swelling kinetics in the hydrogel, and the latter governs the mechanical equilibrium of the bilayer system to swelling-induced stresses. The Newmark approximation and Generalized alpha method were used for time domain discretization and solving the semi-discrete equation. The study also investigated vital parameters influencing bilayer swelling kinetics using semi-analytical and FEM approaches. This section will summarize the findings and their implications for future research.

The design and optimization of the bilayers based on hydrogel for various applications can benefit from the significant implications of our research findings. Our study has shown that the equilibration time of hydrogels can be notably decreased by adjusting the buffer concentration and hydrogel thickness. Furthermore, it was found that the stress discontinuity at the interface of the bilayer can be improved to prevent interface rupture. Our results also suggest that the network’s final slope and water absorption can be deliberately altered for biosensors or other applications. In summary, our study has yielded a comprehensive understanding of the complex behavior of bilayers based on hydrogel, thus advancing our knowledge of this complex system and suggesting an effective tool for designing various types of bilayers. The capacity of the model to design responsive bilayers based on hydrogel for different fields of use could be explored in future research, thereby opening up new avenues for research in these areas.

## 4. Theoretical Background

**Figure 12:**
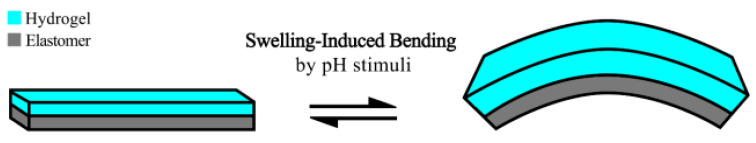
Bilayer actuation in response to pH stimuli: At pH conditions away from the pKa of the hydrogel functional groups, the bilayer bends due to swelling of the hydrogel layer.

A bilayer consists of two materials: a hydrogel that responds to changes in pH and a compressible elastomer layer, as illustrated in Figure 12. This problem involves coupling two distinct physical phenomena: electrochemistry and mechanics. Firstly, the model used to describe the time-evolving behavior of the hydrogel layer in response to pH stimuli is reviewed. Secondly, the kinematics of the bilayer’s finite deformation under bending, which is a critical component in developing the proposed semi-analytical solution, are presented.

### 4.1. Electrochemical Field

**Figure 13:**
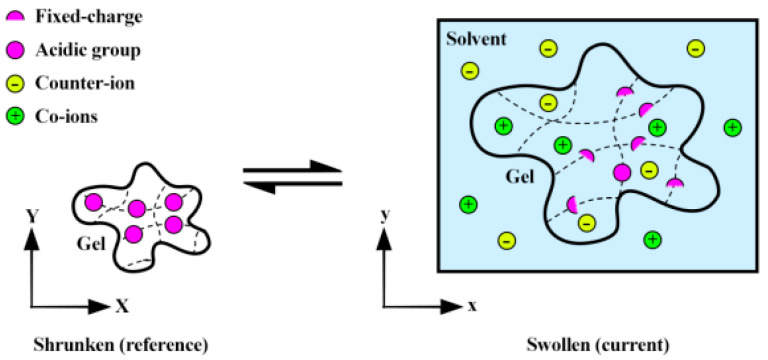
A pH-responsive hydrogel in its dry state (reference) and its swollen state (current) in response to external pH stimuli, the network contains acidic groups, co-ions, counter-ions, and fixed charges [17].

When a hydrogel with acidic functional groups (e.g., carboxylic acid) encounters a higher pH environment, the functional groups can deprotonate, releasing hydrogen ions and generating a negative charge on the polymer network, as illustrated in Figure 13. Conversely, when a hydrogel with primary functional groups (e.g., amino groups) encounters a lower pH environment, the functional groups can protonate, consuming hydrogen ions and generating a positive charge on the polymer network. Due to this charge, the polymer chains start repelling each other, creating an osmotic pressure that draws water into the hydrogel. This reversible swelling behavior is utilized for swelling-induced bending, as shown in Figure 12.

Given the steep concentration gradient of hydrogen ions resulting from pH fluctuations and their exclusive interaction with fixed charges in the hydrogel network, we limit our analysis to their transport and neglect other ionic species. Hydrogen ions in hydrogels are subject to various physiochemical mechanisms that affect their transport, including the dispersion caused by concentration gradients, fluid flow due to hydrogel deformation, and the influence of electrostatic forces. To accurately model these complex phenomena, a mathematical tool that combines the Nernst–Planck equation for charged ion transport and the Poisson equation for electrical potential is considered [34]. This approach provides a comprehensive framework for analyzing ion transport phenomena with high precision. The Nernst–Planck equation takes the form of [34]

(1) $$\frac{\partial c_{k}}{\partial t}+\nabla \cdot \Gamma_{k}=0.$$

The concentration and flux of the $k_{th}$ ion are denoted by $c_{k}$ and $\Gamma_{k}$, respectively. The flux $\Gamma_{k}$ can be decomposed into three components, namely diffusion, electromigration, and advection expressed through the following,

(2) $$\Gamma_{k}=-\phi {\bar{D}}_{k}\nabla c_{k}-\phi \mu_{k}z_{k}c_{k}\nabla \psi +c_{k}V,$$

where ϕ is the porous fraction of the hydrogel, ${\bar{D}}_{k}$ is the diffusion coefficient of the $k_{th}$ ion, $\mu_{k}$ is the mobility coefficient, $z_{k}$ is the electric charge of the $k_{th}$ ion, ψ is the electrostatic potential, and V is the average fluid velocity relative to the gel structure.

Equation (2) indicates that calculating the ionic flux requires accounting for both the concentration of charged species and the electric field in space. Poisson’s equation describes the electrostatic field that governs the movement of charged particles [34].

(3) $$\nabla \cdot \left(\epsilon \epsilon_{0}\nabla \psi \right)=-\frac{F}{\epsilon \epsilon_{0}}\left(\sum_{i=1}^{N}z_{i}c_{i}+z_{o}c_{o}\right).$$

The notation used in this context distinguishes between ions present inside (i) and outside (o) of the hydrogel. The symbol *ϵ* represents the hydrogel’s relative permittivity, while a vacuum’s permittivity is represented by $\epsilon_{0}$. The Poisson’s equation is normalized in the following manner:

(4) $$\kappa^{-2}\nabla \cdot \left[\nabla \left(\frac{e\psi }{k_{B}T}\right)\right]=-\frac{\left(z_{o}c_{o}+\sum_{i}z_{i}c_{i}\right)}{\sum_{i}z_{i}^{2}c_{i}}.$$

The Debye’s length, which is denoted as $\kappa^{-1}$, is characterized by

(5) $$\kappa^{-1}=\sqrt{\frac{\epsilon \epsilon_{0}k_{B}T}{2e^{2}I}},$$

where $I=\frac{1}{2}\underset{o}{\sum }z_{o}^{2}c_{o}$ characterizes the strength of the surrounding ionic solution.

Ionizable acidic groups in pH-sensitive hydrogels give rise to fixed charges and mobile ions, forming an electrical double layer in the domain. This layer surrounds the hydrogel and scales with Debye’s length, which is in the nanoscale range [17]. The behavior of smaller hydrogels can be strongly influenced by this layer, while for larger macroscopic hydrogels that are several micrometers or more in size, the left-hand side of Equation (4) can generally be ignored outside of the double layer. Consequently, the electromigration flux of Equation (2) is negligible inside macroscopic hydrogels.

Due to the geometry of the system and the presence of pH stimuli solely on the outer radial surface of the hydrogel, the flux of hydrogen ions is restricted to the radial direction.

(6) $$\Gamma_{k}=-\phi \frac{{\bar{D}}_{k}}{r\prime }\frac{\partial c_{k}}{\partial r}\hat{r}.$$

To simplify the notation, differentiation with respect to the referential coordinate ($X_{2}$) is denoted by the prime symbol. The porosity of the gel is defined according to a model proposed by [17].

(7) $$\phi =\frac{H}{1+H},$$

where H represents the fraction of water that the hydrogel has absorbed. Ion transport is restricted to the fluid-filled regions of the gel, and the presence of polymer chains can impede the movement of mobile ions by lengthening their travel paths. To model this effect, an obstacle model can be applied to relate the diffusion rate within the gel to the diffusion rate in the surrounding aqueous solution [31].

(8) $${\bar{D}}_{k}=D_{k}{\left(\frac{H}{2+H}\right)}^{2}.$$

When a bilayer hydrogel is immersed in a pH solution, ion transport is mainly characterized by movement in the radial direction. This leads to the development of a one-dimensional continuity equation that effectively captures this behavior, which can be formulated as follows:

(9) $$\frac{\partial }{\partial t}\left(Hc_{k}+Hc_{k}^{b}\right)=-\frac{\partial \left(\alpha \Gamma_{k}\right)}{\partial X_{2}},$$

where $c_{k}^{b}$ is the concentration of the $k_{th}$ ion that can bind reversibly to the polymer fixed charges, $X_{2}$ is the Lagrangian coordinate system, and α is used to normalize the current area of the hydrogel to its initial value. Since the model is one-dimensional, α remains fixed throughout the analysis (α=1). The hydrogel’s chemical reactions facilitate the determination of the reversible binding concentration of ion k to the polymer chains ($c_{k}^{b}$) through the following equation [17]:

(10) $$c_{k}^{b}=\frac{c_{mo}^{s}}{H}\left(\frac{c_{k}}{K+c_{k}}\right),$$

where $c_{mo}^{s}$ is the total concentration of ionizable groups in the gel before swelling. In the hydrogel, only the hydrogen ions interact with the fixed charges of the polymer, causing their concentration to respond sharply to changes in pH. Thus, hydrogen ion migration is the sole species considered in the subsequent analysis.

Buffer solutions significantly influence the swelling behavior of hydrogels through mechanisms such as ion-exchange interactions, pH sensitivity, electrolyte effects, and chemical interactions [35]. According to Azeem, Islam, Rizwan, Rasool, Gul, Khan, Khan, and Rasheed [32], free swelling experiments indicate that hydrogels swell more rapidly in buffered solutions. While buffer solutions can affect various polymer characteristics, their influence on hydrogen ion transport is considered by an extended continuity equation that incorporates additional components as follows [31]:

(11) $$\frac{\partial }{\partial t}\left(Hc_{H}+Hc_{H}^{b}\right)+\frac{\partial }{\partial t}\left(Hc_{HB}\right)=-\frac{\partial \left(\Gamma_{H}\right)}{\partial X_{2}}-\frac{\partial \left(\Gamma_{HB}\right)}{\partial X_{2}},$$

in which $c_{HB}$ indicates the concentration of hydrogen ions bound to the buffer, and $\Gamma_{HB}$ is the flux of hydrogen ions bound to the buffer. The concentration of hydrogen ions bound to the buffer ($c_{HB}$) is expressed in terms of $c_{H}$ by an isotherm reaction, using:

(12) $$c_{HB}=\frac{c_{H}c_{T}}{K_{B}+c_{H}},$$

where $K_{B}$ indicates the buffer’s dissociation constant and $c_{T}$ is the total buffer concentration computed as $c_{T}=c_{B^{-}}+c_{HB}$. The flux of the buffer is related to the flux of the hydrogen ions as [31]

(13) $$\Gamma_{HB}=\Gamma_{H}\left(\frac{{\bar{D}}_{HB}}{{\bar{D}}_{H}}\frac{c_{T}}{K_{B}+c_{H}}\right),$$

where ${\bar{D}}_{HB}$ represents the diffusion coefficient of the buffer. Employing Equations (10), (12) and (13), Equation (11) can be recast as

(14) $$\frac{\partial }{\partial t}\left(Hc_{H}+\frac{c_{mo}^{s}c_{k}}{K+c_{k}}+H\frac{c_{T}c_{H}}{K_{B}+c_{H}}\right)=\frac{\partial }{\partial X_{2}}\left[\frac{1}{r\prime }\left(\frac{H}{1+H}\right)\left(1+\frac{{\bar{D}}_{HB}}{{\bar{D}}_{H}}\frac{c_{T}}{K_{B}+c_{H}}\right)\left({\bar{D}}_{H}\frac{\partial c_{H}}{\partial r}\right)\right].$$

### 4.2. Finite Bending of Bilayer Kinematics

**Figure 14:**
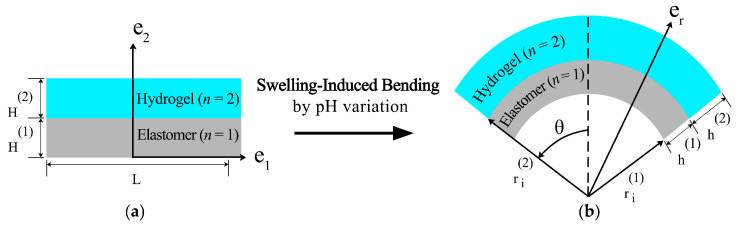
Two distinct bilayer configurations: (**a**) reference configuration with elastomer (n = 1) at the bottom and hydrogel (n = 2) on top, and (**b**) current configuration under external stimuli.

This section presents the necessary mathematical equations for analyzing the deflection due to the bending of a bilayer composed of a hydrogel layer and a compressible elastomer layer. The initial undeformed state of the material is described using a Cartesian coordinate system with three variables: $X_{1}$, $X_{2}$, and $X_{3}$. However, to determine the shape of the material when undergoing bending, a cylindrical coordinate system is required, as suggested by Rajan and Arockiarajan [36]. This transformation necessitates a mathematical conversion. The domain of the undeformed configuration is defined as follows:

(15) $$X_{1}\in \left[-\frac{L}{2},+\frac{L}{2}\right],\;\;X_{2}\in \left[0,H^{\left(1\right)}+H^{\left(2\right)}\right],\;\;X_{3}\in \left(-\infty ,+\infty \right),$$

where L and $H^{\left(n\right)}$ are the length and thickness of each layer, respectively. It is important to note that the initial thickness of the structure is equal to the sum of the thicknesses of each layer, i.e., $H=H^{\left(1\right)}+H^{\left(2\right)}$. Figure 14 provides an illustration that helps readers better understand the material’s coordination.

Using cylindrical coordinates x=(r, θ, z) to describe the system, the position of any given point $x^{\left(n\right)}$ in the system at the current time t can be expressed as [2]:

(16) $$x^{\left(n\right)}=r^{\left(n\right)}\left(X_{2},t\right)e_{r}+\theta^{\left(n\right)}\left(X_{1},t\right)e_{\theta }+z^{\left(n\right)}\left(X_{3},t\right)e_{z}.$$

Confined with

(17) $$r^{\left(1\right)}\in \left[r_{i}^{\left(1\right)},r_{i}^{\left(1\right)}+h^{\left(1\right)}\right],r^{\left(2\right)}\in \left[r_{i}^{\left(2\right)},r_{i}^{\left(2\right)}+h^{\left(2\right)}\right],\;\theta^{\left(n\right)}\in \left[-\bar{\theta },+\bar{\theta }\right],\;z^{\left(n\right)}\in \left(-\infty ,+\infty \right).$$

The base vectors of the cylindrical coordinate system are represented by $e_{i}$ (i=r, θ, z). Each layer’s current inner radius and thickness are denoted by $r_{i}^{\left(n\right)}$ and $h^{\left(n\right)}$, respectively. Notably, $r_{i}^{\left(2\right)}$ is identical to $r_{i}^{\left(1\right)}+h^{\left(1\right)}$.

The deformation gradient for the hydrogel and elastomer layers is calculated separately using the approach suggested by Bakhtiyari, Baghani, and Sohrabpour [2].

(18) $$F^{\left(n\right)}=\frac{dr^{\left(n\right)}\left(X_{2},t\right)}{dX_{2}}e_{r}⊗e_{2}+r^{\left(n\right)}\left(X_{2},t\right)\frac{d\theta^{\left(n\right)}\left(X_{1},t\right)}{dX_{1}}e_{\theta }⊗e_{1}+e_{z}⊗e_{3}.$$

The symbol denotes a dyadic product, while the definition of deformation gradient relies on the plane-strain assumption. Using the obtained deformation gradients, the principal stretches can be determined through the following:

(19) $$\lambda_{r}^{\left(n\right)}=\frac{dr^{\left(n\right)}\left(X_{2},t\right)}{dX_{2}},\;\;\lambda_{\theta }^{\left(n\right)}=r^{\left(n\right)}\left(X_{2},t\right)\frac{d\theta^{\left(n\right)}\left(X_{1},t\right)}{dX_{1}},\;\;\lambda_{z}^{\left(n\right)}=1.$$

The tangential component of the position vector in the cylindrical coordinate is distributed linearly and can be represented by the equation $\theta^{\left(n\right)}=2\bar{\theta }X_{1}/L$. Thus, for every layer, the tangential principal stretch can be established using the following:

(20) $$\lambda_{r}^{\left(n\right)}=\frac{2\bar{\theta }}{L}r^{\left(n\right)}\left(X_{2},t\right).$$

To accurately quantify the deformation of the hydrogel and elastomeric layers, it is necessary to compute the Cauchy–Green strain tensor using $C=F^{T}F$. This tensor plays a critical role in characterizing the deformation of these layers and is a distinguishing feature of both materials.

(21) $$C^{\left(n\right)}=\mathrm{diag}\;\left({\left(\frac{dr^{\left(n\right)}\left(X_{2},t\right)}{dX_{2}}\right)}^{2},\frac{4{\bar{\theta }}^{2}}{L^{2}}r^{\left(n\right)}{\left(X_{2},t\right)}^{2}\;,1\right).$$

The tensors provide the first and third invariants that are required for the subsequent steps in the analysis, as follows:

(22) $$\begin{array}{c} I_{1}^{\left(n\right)}=1+{\left(\frac{dr^{\left(n\right)}\left(X_{2},t\right)}{dX_{2}}\right)}^{2}+\frac{4{\bar{\theta }}^{2}}{L^{2}}r^{\left(n\right)}{\left(X_{2},t\right)}^{2},\\ I_{3}^{\left(n\right)}=\frac{4{\bar{\theta }}^{2}}{L^{2}}{\left(\frac{dr^{\left(n\right)}\left(X_{2},t\right)}{dX_{2}}\right)}^{2}r^{\left(n\right)}{\left(X_{2},t\right)}^{2}. \end{array}$$

### 4.3. Governing Equations

Earlier in this study, the material model and kinematics of the bilayer were addressed. Now, a transient semi-analytical solution for studying the finite bending of the hydrogel-based bilayer is established. In order to model the finite deformation of the bilayer materials, a neo-Hookean-based compressible elastic energy will be applied [37]. This energy is particularly effective at accounting for the highly non-linear manner of the dependent solid fraction elasticity.

(23) $$W_{net}{}^{\left(n\right)}=\frac{1}{8}M^{\left(n\right)}{ln}^{2}\left[\det\left(C^{\left(n\right)}\right)\right]+\frac{1}{2}G^{\left(n\right)}\left[\mathrm{tr}\left(C^{\left(n\right)}\right)-3\det{\left(C^{\left(n\right)}\right)}^{\frac{1}{3}}\right].$$

$M^{\left(n\right)}$ and $G^{\left(n\right)}$ are denoted as each layer’s hyperelastic constants of the strain energy function. Using the as-mentioned energy function, it becomes feasible to determine the stress distribution of each layer by following the subsequent procedure:

(24) $$\sigma^{\left(n\right)}=\frac{1}{J}F^{\left(n\right)}\cdot \frac{\partial W_{net}{}^{\left(n\right)}}{\partial F}\cdot {F^{\left(n\right)}}^{T}+\sigma_{ext}{}^{\left(n\right)}.$$

By utilizing Equations (23) and (24), the stress constitutive equations can be reformulated as follows:

(25) $$\begin{array}{c} \sigma_{rr}{}^{\left(n\right)}=\frac{ML}{4{r^{\prime }}^{\left(n\right)}r^{\left(n\right)}\theta }ln\left(\frac{4{{r^{\prime }}^{\left(n\right)}}^{2}\theta^{2}{r^{\left(n\right)}}^{2}}{L^{2}}\right)-\frac{G}{2}\sqrt[{3}]{\frac{4L}{{r^{\prime }}^{\left(n\right)}r^{\left(n\right)}\theta }}+\frac{GL{r^{\prime }}^{\left(n\right)}}{2r^{\left(n\right)}\theta },\\ \sigma_{\theta \theta }{}^{\left(n\right)}=\frac{ML}{4r\prime^{\left(n\right)}r^{\left(n\right)}\theta }ln\left(\frac{4{{r^{\prime }}^{\left(n\right)}}^{2}\theta^{2}{r^{\left(n\right)}}^{2}}{L^{2}}\right)-\frac{G}{2}\sqrt[{3}]{\frac{4L}{r\prime^{\left(n\right)}r^{\left(n\right)}\theta }}+\frac{2Gr^{\left(n\right)}\theta }{Lr\prime^{\left(n\right)}},\\ \sigma_{zz}{}^{\left(n\right)}=\frac{ML}{4r\prime^{\left(n\right)}r^{\left(n\right)}\theta }ln\left(\frac{4{{r^{\prime }}^{\left(n\right)}}^{2}\theta^{2}{r^{\left(n\right)}}^{2}}{L^{2}}\right)-\frac{G}{2}\sqrt[{3}]{\frac{4L}{r\prime^{\left(n\right)}r^{\left(n\right)}\theta }}+\frac{GL}{2r\prime^{\left(n\right)}r^{\left(n\right)}\theta }. \end{array}$$

The external stress term of (24) is only non-zero for the hydrogel layer when it expands due to pH stimuli. Therefore, the osmotic pressure of $\Pi =\Pi_{mix}+\Pi_{ion}$ is considered a volumetric external stress in principal directions, as follows:

(26) $$\sigma_{ext}{}^{\left(2\right)}=\mathrm{diag}\left(\Pi ,\;\Pi ,\;\Pi \right).$$

Within the gel network, the osmotic pressure (Π) can be expressed as the sum of two distinct terms: $\Pi_{mix}$, which arises from the mixing of the solvent and the network, and $\Pi_{ion}$, which arises due to a difference in the concentration of ions present within the gel as compared to the external solution. According to Kurnia et al. [38], the available evidence suggests that $\Pi_{ion}$ is the primary contributor, mainly originating from the fixed charges and salt concentration within the external solution. Other ionic species have a minimal effect on osmosis due to their low concentration and smaller size order of magnitude. The application of the laws of thermodynamics yields the following conclusion [39]:

(27) $$\Pi \approx \Pi_{ion}=RT\Sigma \left(c_{k}-c_{k0}\right),$$

where k ∈ {f,+,−}, R is the ideal gas constant, and T is the absolute temperature. The concentration of co-ions and counter-ions within the hydrogel can be determined using the Donnan theory [17]. According to this theory, the activity coefficient of the mobile ions in the hydrogel is assumed to be equal to that of the surrounding solution.

(28) $$c_{\pm }=\frac{1}{2}\left(\mp c_{fix}+\sqrt{c_{fix}{}^{2}+4c_{salt}^{2}}\right).$$

An equation that relates the deformation and the absorbed fluid volume within the hydrogel network is assumed as follows:

(29) $$H=J\left(1+H_{0}\right)-1,$$

where J denotes the deformation Jacobian, while $H_{0}$ and H are the initial and current absorbed water faction (hydration) of the hydrogel network, respectively.

Kurnia, Birgersson, and Mujumdar [38] suggested that the hydrogel’s deformation takes place at a slower rate than the transfer of ions, thereby a static approach is suitable for the analysis. This means that the equilibrium equation in a cylindrical coordinate system can be rewritten to consider the preservation of linear momentum in the absence of external loads.

(30) $$r^{\left(n\right)}\left(X_{2},t\right)\frac{\partial \sigma_{rr}{}^{\left(n\right)}}{\partial X_{2}}+\frac{\partial r^{\left(n\right)}\left(X_{2},t\right)}{\partial X_{2}}\left(\sigma_{rr}{}^{\left(n\right)}\left(X_{2}\right)-\sigma_{\theta \theta }{}^{\left(n\right)}\left(X_{2}\right)\right)=0.$$

### 4.4. Boundary-Value Problem

This section offers a comprehensive approach to solving the boundary value problem (BVP), which involves solving both a partial differential equation (Equation (14)) and an ordinary differential equation (Equation (30)) simultaneously. Proper boundaries and initial conditions are presented in Table 1, enabling a solution to this challenging problem.

From a chemical perspective, swelling-induced bending of a hydrogel-based bilayer involves two stages. Firstly, the hydrogel is equilibrated in a solvent with low pH (i.e., pH=3). According to experimental work by De, Aluru, Johnson, Crone, Beebe, and Moore [31], this equilibration level corresponds to hydration of 20% ($H_{0}=0.2$). We assume a uniform initial concentration of $c_{H0}$ across the hydrogel domain in the first stage to ensure consistency. Subsequently, the hydrogel is exposed to a surrounding solvent with an elevated pH value (pH=6), which induces its swelling. Boundary conditions are used to model the swelling process of the hydrogel. Specifically, a Dirichlet boundary condition is imposed on the outer surface of the hydrogel to define the concentration of $c_{H}$. In contrast, a Neumann boundary condition is used at the bilayer interface to enforce zero flux during swelling.

From a mechanical perspective, the outer and inner surfaces of the hydrogel-based bilayer do not experience traction during swelling, causing the radial Cauchy stress to approach zero. To ensure this, a boundary condition is applied to the surfaces. Additionally, the continuity of bonding between layers at the bilayer interface is maintained by enforcing a matching condition, which is also imposed as a boundary constraint.

Since the bending of the bilayer is solely caused by the hydrogel swelling and mechanical stiffness mismatch between the layers, the moments at the side surfaces must cancel out, resulting in an equilibrium of moments around the bilayer. Consequently:

(31) $$\int_{0}^{H^{\left(1\right)}}r^{\left(1\right)}\left(X_{2},t\right)\sigma_{\theta \theta }^{\left(1\right)}\frac{\partial r^{\left(1\right)}\left(X_{2},t\right)}{\partial X_{2}}dX_{2}+\int_{H^{\left(1\right)}}^{H^{\left(1\right)}+H^{\left(2\right)}}r^{\left(2\right)}\left(X_{2},t\right)\sigma_{\theta \theta }^{\left(2\right)}\frac{\partial r^{\left(2\right)}\left(X_{2},t\right)}{\partial X_{2}}dX_{2}=0.$$

The chemical properties utilized in the present simulations, detailed in Table 2, are based on prior research [31]. While the hyper-elastic material constants of the hydrogel are determined through a fitting curve technique, the elastic behavior of the elastomer is also determined by the Wilson hyperelastic strain energy [17], with material constants selected based on practical considerations. This enables the choice of a broad range of compressible soft polymers suitable for various applications in tissue engineering and bioelectronics. Examples of such materials include PDMS and polyurethane (PU), among others.

### 4.5. Limiting Assumptions of the Solution

- The main focus of this study is on macroscopic hydrogels, which are assumed to satisfy electro-neutrality conditions, given Debye’s length in the nanometer range. The hypotheses, assumptions, and theories are chosen and refined with care to ensure that the conclusions drawn are specific and targeted;
- In this study, chemical reactions are assumed to be reversible and infinitely fast, resulting in the instantaneous attainment of equilibrium in the time dimension. Moreover, the activity coefficient of mobile ions in the hydrogel is considered equivalent to that of the equilibrium solution;
- Rather than using a multi-phase approach, we treat the hydrogel and solvent as a single-phase domain. While this simplified model may not fully capture all the complexities of hydrogel behavior, it enables more efficient and practical analysis of the system under investigation. Even with this simplified approach, valuable insights into the behavior of hydrogels and their potential applications can still be gained;
- The effect of fluid dynamics in the hydrogel domain is overlooked in this study due to its negligible impact [31]. Instead, diffusion is identified as the hydrogel’s primary cause of ion migration. Therefore, it is appropriate to assume a quasi-static condition to study the mechanical response;
- According to Kurnia, Birgersson, and Mujumdar [38], osmotic pressure primarily influences the swelling behavior of pH-responsive polymers when no external electric field is present. As a result, we neglect the contribution of electrostatic forces and the tendency of the polymer network to mix with the surrounding solution in this analysis.

## 5. Computational Framework

The governing equations of the system are composed of two dependent variables, namely current radius $r^{\left(n\right)}$ and the current concentration of the hydrogen field, $c_{H}$, which are interdependent and undergo simultaneous changes over time. Discretization using the finite element method (FEM) allows us to express the chemical and mechanical fields of the system as a system of two equations in matrix form. To be specific, we can represent the chemical and mechanical fields separately using two matrices in the system.

(32) $$D\dot{c}+K_{d}c=f_{d},$$

(33) $$K^{\left(n\right)}{}_{m}r^{\left(n\right)}=f^{\left(n\right)}{}_{m}.$$

Equation (32) comprises the damping D matrix, diffusion $K_{d}$, and the vector of flux $f_{d}$ of Equation (14). On the other hand, Equation (33) is composed of a matrix of stiffness$\;K^{\left(n\right)}{}_{m}$, and the vector of force $f^{\left(n\right)}{}_{m}$ of Equation (30).

Applying FEM as a solution in the time domain is viable, but it can come with a high computational cost. An alternative approach is the derivation of the Newmark approximation for time domain concentration,

(34) $${\dot{c}}_{n+1}\left(c_{n+1}\right)=\frac{\gamma }{\beta }\left(\frac{c_{n+1}-c_{n}}{\Delta t}\right)-\frac{\gamma -\beta }{\beta }{\dot{c}}_{n}.$$

The Newmark empirical constants, γ and β, can be utilized in the half-time approximation of c and $\dot{c}$. This approximation allows for the solution of $c_{n+1}$ as the only unknown in the time domain; the semi-discrete form of Equation (32) can be employed with the generalized alpha method to solve for as follows after substituting Equation (34).

(35) $$\left[D\frac{\gamma \left(1-\alpha_{f}\right)}{\beta \Delta t}+K\left(1-\alpha_{f}\right)\right]c_{n+1}=f_{n+1-\alpha_{f}}-K\alpha_{f}c_{n}+D\left[\frac{\gamma \left(1-\alpha_{f}\right)}{\beta \Delta t}c_{n}+\left(\frac{\gamma -\gamma \alpha_{f}-\beta }{\beta }\right){\dot{c}}_{n}\right].$$

According to this method, an iterative technique is used to apply it. In the first iteration, an initial guess is used for $\bar{\theta }$. The first iteration is solved, and then the net tangential moment of Equation (31) is assumed to be zero for any strip cross-section. An optimization technique can then be used to calculate the initial guess for the half-angle in subsequent iterations.

## 6. Materials and Methods

The present study utilized a semi-analytical approach to predict pH-sensitive hydrogel behavior. To validate the accuracy of this approach, previous experimental results were utilized [31,33]. The experimented hydrogels comprised homogeneous copolymers (HEMA and acrylic acid) crosslinked with a 1% diacrylate. These hydrogels displayed pH sensitivity due to carboxyl groups attached to the polymer chains. The current study focuses primarily on the swelling mechanism attributed to the propulsion of fixed charges arising from the dissociation of functional groups. Although hydrogen bonding interactions between the undissociated carboxyl and hydroxylic groups may affect the swelling behavior by strengthening intramolecular attractions within the copolymer, the presented model disregards such hydrogen bonding interactions [31,40,41]. The validity and reliability of the proposed approach in the current study were confirmed by comparing the predictions of the semi-analytical method with the experimental findings.

The semi-analytical proposed in this study utilizes the finite element method (FEM) for discretization and solves equations. It includes the application of the Newmark approximation for time domain concentration and the generalized alpha method for iterative solution.

## Figures and Tables

**Table 1:** Conditions are needed to solve the boundary value problem of bilayer bending resulting from pH-triggered swelling.

Scope	Boundary Conditions	Initial Conditions
**Chemical**	$\frac{\partial c_{H}}{\partial R}=0\;at\;X_{2}=H^{\left(1\right)}$	$c_{H}=10^{-3}\left[M\right]$
	$c_{H}=10^{-6}\left[M\right]\;at\;X_{2}=H^{\left(1\right)}+H^{\left(2\right)}$	
**Mechanical**	$\sigma_{rr}=0\;at\;X_{2}=0$	$H_{0}=0.2$
	$\sigma_{rr}=0\;at\;X_{2}=H^{\left(1\right)}+H^{\left(2\right)}$	
	$r^{\left(1\right)}\left(X_{2},t\right)=r^{\left(2\right)}\left(X_{2},t\right)\;at\;X_{2}=H^{\left(1\right)}$	

**Table 2:** Chemical and mechanical parameters utilized for bilayer bending resulting from pH-triggered swelling.

Physics	Parameter	Hydrogel	Elastomer
**Mechanical**	M	13.27 Mpa	17.25 Mpa
	G	0.227 Mpa	0.273 Mpa
**Chemical**	${\bar{D}}_{H}$	$9.3\times 10^{-9}\frac{m^{2}}{s}$	
	${\bar{D}}_{HB}$	$8.79\times 10^{-10}\frac{m^{2}}{s}$	
	$c_{mo}^{s}$	1800 mM	
	K	$10^{-2.0}\;\mathrm{mM}$	
	$K_{B}$	$10^{-5}\;\mathrm{mM}$	

## Data Availability

We confirm that all the relevant data and results from our study have been included in the paper itself. Therefore, there is no additional data to be shared or archived.
